# Prognostic significance of quantitative measures of myocardial infarct pathology using native T1 mapping, in survivors of ST-elevation myocardial infarction

**DOI:** 10.1186/1532-429X-17-S1-O53

**Published:** 2015-02-03

**Authors:** David Carrick, Caroline Haig, Samuli M Rauhalammi, Nadeem Ahmed, Ify Mordi, Margaret McEntegart, Mark Petrie, Hany Eteiba, Stuart Hood, Stuart Watkins, Mitchell Lindsay, Ahmed Marous, Aleksandra Radjenovic, Ian Ford, Niko Tzemos, Keith G Oldroyd, Colin Berry

**Affiliations:** 1 Golden Jubilee National Hospital, Clydebank, UK; 2 Institute of Cardiovascular and Medical Sciences, University of Glasgow, Glasgow, UK; 3 Robertson Center for Biostatistics, University of Glasgow, Glasgow, UK

## Background

Myocardial longitudinal relaxation time (T1, ms) is a fundamental magnetic property of tissue that is related to water content and mobility. The pathophysiological and prognostic importance of native myocardial T1 values in acute STEMI patients is unknown. We aimed to assess the clinical significance of infarct tissue characteristics using native T1 cardiac magnetic resonance (CMR).

## Methods

We performed a prospective single center cohort study in reperfused STEMI patients who underwent CMR 2 days and 6 months post-MI. Native T1 CMR (MOLLI investigational prototype sequence: 3 (3) 3 (3) 5) was measured in myocardial regions-of-interest. The area-at-risk and infarct territory were depicted with T1 mapping and late gadolinium contrast enhancement imaging, respectively. All-cause death or heart failure hospitalization was a pre-specified outcome that was assessed during follow-up.

## Results

300 STEMI patients (mean±SD age 59±12 years, 74% male, 114 with anterior STEMI) gave informed consent and had CMR (14 July 2011 - 22 November 2012). Of these, 288 STEMI patients had evaluable T1 maps and follow-up assessments (median duration 845 days). Infarct size was 18 ±14% of left ventricular mass. Microvascular obstruction occurred in 160 (55.6%) patients. Native T1 within the area-at-risk (1097 ±52 ms) was higher than in the remote zone (961 ±25 ms; p<0.01) or infarct core (996.9 (57.3); p<0.01). In multivariable linear regression, native T1 in the area-at-risk was independently and negatively associated with age, initial systolic blood pressure and TIMI coronary flow grade at presentation (all p<0.05), independent of LVEF, left ventricular end-diastolic volume (LVEDV) or infarct size. Similar findings were observed for T1 core values, which were also independently associated with Killip class.

At 6 months, LVEDV change was available for 262 patients and was increased by ≥20% in 30 (11.5%) patients of whom 23 (76.7%) had microvascular obstruction at baseline. T1 in the infarct core was a multivariable predictor of adverse remodeling (-0.01 (-0.02, -0.00); p=0.048).

288 (100%) patients had longer term follow-up completed. The median duration of follow-up was 845 days. Thirty (10.4%) patients died or experienced a heart failure event and 13 (4.5%) of these patients died or experienced a heart failure hospitalization post-discharge. Native T1 in the area-at-risk was a predictor of all-cause death or heart failure post-discharge (hazard ratio 0.985, 95% CI 0.973, 0.997; p=0.012) including after adjustment for LVEF at baseline (p=0.018) and LVEDV at baseline (p=0.013).

## Conclusions

In acute STEMI patients, infarct zone tissue characteristics predict left ventricular remodeling and long-term health outcomes, independent of infarct size and LV function.

## Funding

N/A.

**Figure 1:**
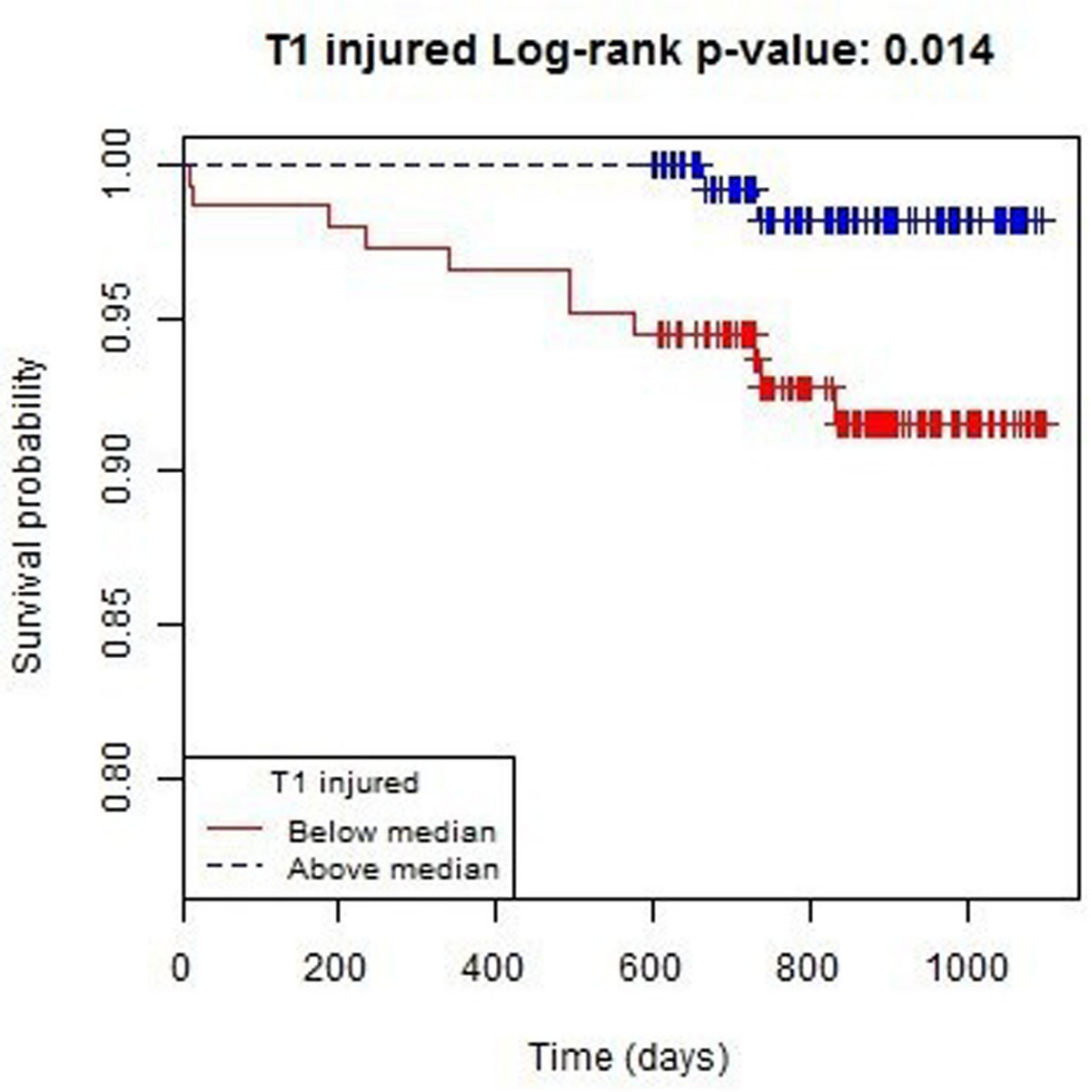
Kaplan-Meier survival plot (n = 288) for all-cause death or heart failure hospitalisation by T1 injured median.

